# Back to the future: breast surgery with tumescent local anesthesia (TLA)?

**DOI:** 10.1007/s00404-023-06938-5

**Published:** 2023-03-06

**Authors:** B. Boeer, G. Helms, J. Pasternak, C. Roehm, L. Kofler, H. M. Haefner, M. Moehrle, E. Heim, H. Fischer, S. Y. Brucker, M. Hahn

**Affiliations:** 1 Department of Women’s Health, University Hospital Tuebingen, Calwerstrasse 7, 72076 Tuebingen, Germany; 2 Department of Dermatology, University Hospital Tuebingen, Tuebingen, Germany; 3 Praxisklinik Haut Und Venen, Tuebingen, Germany; 4 Department of Anesthesiology and Intensive Care Medicine, University Hospital Tuebingen, Tuebingen, Germany

**Keywords:** Tumescent local anesthesia, TLA, Breast surgery

## Abstract

**Purpose:**

Breast surgery is usually performed under general anesthesia. Tumescent local anesthesia (TLA) offers the possibility to anesthetize large areas with highly diluted local anesthetic.

**Methods:**

In this paper, the implementation, and experiences with TLA in the field of breast surgery are discussed.

**Conclusion:**

For carefully selected indications, breast surgery in TLA represents an alternative to ITN.

## What does this study add to the clinical work

**Table:** 

Most common breast surgeries can be performed under tumescent local anesthesia and therefore should be offered as an alternative to general anesthesia. This article provides insight into this anesthetic technique in the field of breast surgery.	

## Introduction

Breast surgery is usually performed under general anesthesia. Rarely or in special situations, these procedures are performed under local anesthesia—especially in multimorbid or elderly patients [1–3].

In contrast to common local anesthesia (LA), in which the maximum dose is reached quickly, and only small areas are anesthetized, tumescent local anesthesia (TLA) allows larger areas to be anesthetized by direct infiltration of considerable amounts of a highly diluted local anesthetic.

The Latin word “tumescere” means “to swell”—the typical aspect of the tissue after infiltration.

The first to describe this infiltration anesthesia for various operations—using cocaine as an analgesic—was the surgeon C.L. Schleich in 1892 [4]. However, due to the emergence of ether anesthesia at that time, the idea was not pursued further and was not revived until 1987 by the American plastic surgeon Jeffrey Klein, who successfully used lidocaine instead of cocaine for liposuction [5]. Over time, the dose of local anesthetic was progressively reduced while maintaining the same effect, and automatic infiltration pump systems facilitated the treatment of large areas. Thus, Gerhard Sattler introduced the method of liposuction with a roller pump in Germany in 1994 [6].

Breuninger developed subcutaneous infusion anesthesia (SIA) with flow- and volume-controlled infusion devices in 1998 [7]; he used Ringer's solution as a base without further addition of sodium bicarbonate and introduced the long-term local anesthetic ropivacaine, on the market since 1994, into the local anesthetic mixture [8].

Due to its long-lasting effect, local anesthesia can be separated in time from the surgical procedure and lasts longer postoperatively.

TLA is now used interdisciplinarily and across the surgical spectrum, particularly in dermatologic and plastic surgery or surgery of young children [9].

The use of TLA in the field of senology consists mainly in the addition of tumescent solution in the breast to improve dissection, decrease blood loss, and reduce complications from electrocautery [10–12]–under general anesthesia.

The first description of mastectomy with TLA was by Worland in 1996 [13]. Since then, many studies, reviews, and meta-analyses have shown that preoperative infiltration of a tumescent solution—usually consisting of epinephrine and balanced full electrolyte solution and local anesthetic (usually lidocaine)—reduces perioperative blood loss and postoperative pain in mastectomies with or without reconstruction. In reduction mammoplasty, it can also shorten the operative time [14–17].

There are few publications with small numbers of cases, mostly older patients at high risk of anesthesia (ASA III-IV), describing breast surgery-mostly mastectomies-under local anesthesia alone [2, 3] or TLA [1, 18], always with the addition of sedation (e.g. propofol, midazolam, fentanyl).

Known complications of general anesthesia such as postoperative nausea, vomiting (PONV) or postoperative cognitive dysfunction (POCD) can be avoided, especially in the elderly. Retrospective studies, some with large case numbers, have been published by plastic surgeons on augmentation and reduction mammoplasty for TLA in the outpatient setting [19, 20].

Based on the literature and to our knowledge, no diagnostic or oncoplastic breast surgeries have been routinely performed under TLA. Since various breast surgeries have been performed in TLA at the Department of Women’s Health in Tuebingen since January 2022, this article aims to provide insight into the TLA technique that has been used for breast surgeries.

### Preparations before the operation

Before TLA is started, the patient is informed in detail about the upcoming steps. Routinely, the patient is presented to an anesthesiologist if he or she is > 70 years of age, has an ASA or a NYHA score of ≥ III, or is obese (> BMI 30).

A comfortable environment, empathetic staff, and good communication between surgeon and patient during TLA application and surgery are an important basis for the successful performance of all procedures [21].

Patients should take their usual medications and can drink up to 2 h before surgery. Initially, depending on the planned surgery, all necessary image-guided wire placements are made in our facility. Stereotactic marker wires can be placed after TLA application, so no further local anesthesia is required. In case of breast cancer, Technetium (99mTc) radiolabeled colloids are administered ipsilateral periareolar for later sentinel node biopsy.

**Fig. 1:**
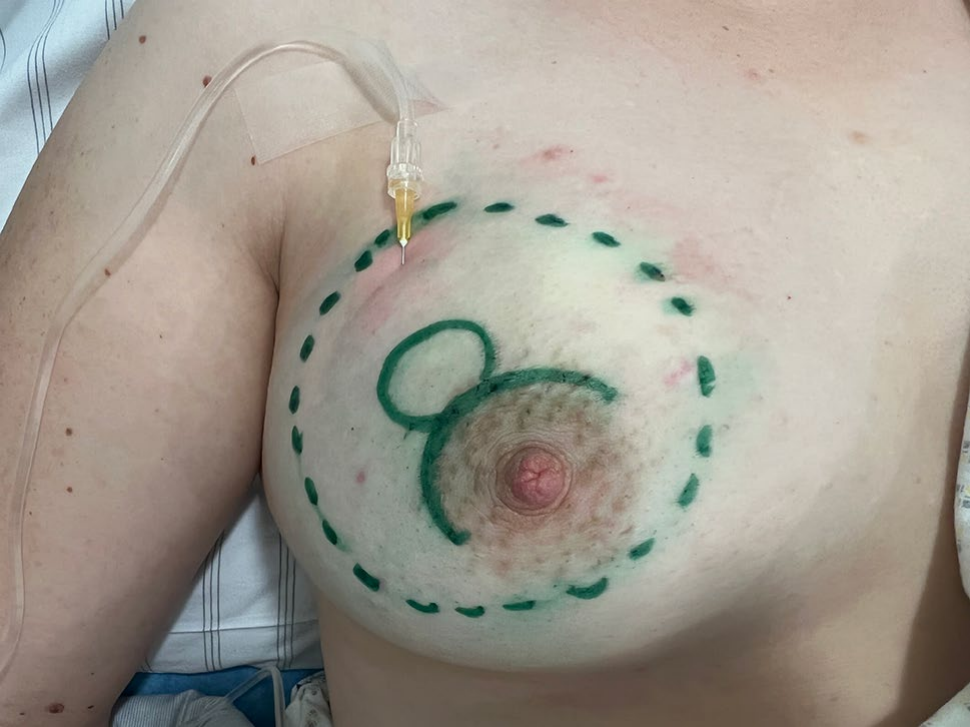
Skin marking of the area to be removed, the area to be anesthetized as well as the planned incision. The infiltrated area becomes pale and tense

Using a pen on the patient’s skin, the surgeon marks the area to be removed and the area to be anesthetized as well as the planned incision (see Fig. 1). Afterwards the surgeon selects the TLA concentration, taking into account the size of the surgical area and the time window (Table 1).

**Table 1:** TLA- solutions using a premanufactured stock solution (ropivacaine 10 mg (1%) + lidocaine 20mg (2%) per 1 ml)

Concentration	Sterofundin (ml)	Stock solution	Epinephrine (1:1000)	Max Dose	Beginning of the effect (min)
0.21%	500 ml	40 ml	0.5 ml	3 ml/kgbw	2–5 min
0.05%	500 ml	10 ml	0.5 ml	12 ml/kgbw	20 min

TLA is then applied by an intern/resident via infusion pump systems or centrifugal pumps with 25- or 27-G needles in an OR prep room.

### Tumescent solutions

There are no general recommendations for the dosage of a TLA—different institutions and situations may use different compositions.

The following points should be considered:

The basis of the tumescent solution is a full electrolyte solution (e.g. Sterofundin ISO), so that the addition of bicarbonate can be dispensed with. As an analgesic, a combination of a short-acting local anesthetic such as lidocaine (lowest risk of inducing methemoglobinemia, max. dose between 35 and 55 mg/kilogram body weight [22]), and ropivacaine as a long-acting local anesthetic is useful [8]. The efficacy lasts for about 15 h postoperatively.

On the one hand, the combination of several local anesthetics with different half-lifes leads to a faster onset and a longer duration of the analgesic effect. On the other hand, toxic effects may be enhanced with higher amounts of free active ingredients due to competitive protein binding [23]. Therefore, when mixing local anesthetics, attention must be paid to the dosage of each component, as it differs from the dosage when used alone [24], taking into account the maximum dose in TLA (Table 1). Regarding pharmacokinetics, several studies have shown that it is possible to safely mix these agents [25]. In these cases, the patient must be informed of the off-label use of the mixture.

Through a cooperation with the University Pharmacy of Tuebingen, the Department of Women’s Health receives a thermally sterilized stock solution prepared in a clean room atmosphere in compliance with hygiene requirements and containing 10 mg ropivacaine (1%) and lidocaine 20 mg (2%) per 1 ml (ph 4; additives: aqua ad inj., hydrochloric acid/sodium hydroxide solution).

On the day of surgery, this stock solution is diluted with whole electrolyte solution according to the desired concentration (see Table 1), which considerably speeds up and simplifies the preparation of TLA [26]. This provides physically stable, easy-to-handle, and ready-to-use TLA solutions at various concentrations that can be used for 24 h. The addition of epinephrine results in vasoconstriction, thus reducing blood loss and prolonging the anesthetic effect. At a dose of 0.5 mg epinephrine, no systemic effect is expected with an overall large infusion volume.

The Department of Women’s Health in Tuebingen works with two different concentrations, depending on the surgical area and volume as well as time window:

- 0.05% TLA solution for large breasts and time lead (0.5 to approx. 2 h) to surgery.
- 0.21% TLA solution for small operations, limited anesthetic area, short lead time and for intraoperative additional analgesia.

### Devices, needles and infiltration speed

Using infusion devices (e.g. Infusomat compact plus B. Braun), gentle application with an infiltration rate of 50–1500 ml/hour is possible—depending on the localization, the extent of the planned intervention, the tissue density or pressure and the needle size [7], taking into account the maximum dose limits (see Table 1).

Because the infusion is semiautomatic, monitoring can be delegated to supporting stuff who can infiltrate multiple patients simultaneously. Thus, the physician does not have to stand next to the patient during this lengthy process. The slow infiltration speed with fine needles (e.g., 27- or 25-G needles) does not usually cause pain in the patient, but rather a feeling of tension.

Since this form of subcutaneous infusion anesthesia is very time-consuming, it is sometimes useful to use additional centrifugal pumps (e.g., Dispenser DP 30). According to the manufacturer, 17 l/h, i.e. 280 ml/min, can be applied via a foot switch. The flow rate can be changed via a rotary switch (0–100%) by reducing the lumen of the silicone tube above the roller. The volume applied depends on the tissue pressure and the cannula used (27G needle: 450–600 ml/h; 25G needle: 800–1000 ml/h; 22G needle: 1500 ml/h). The maximum running speed without discomfort should be determined together with the patient.

If the infiltrated area becomes pale and tense, the needle can be placed elsewhere (Fig. 1).

Initially, TLA is usually applied superficially with a fine needle (25–27 G). If deeper infiltration is required at a later stage (e.g. in the axilla), a non-ground needle should be used to avoid deep injury, as TLA is usually performed without sonographic control.

### Monitoring during TLA

In the run-up to TLA and surgery, the patient is informed in detail about the upcoming steps. Prior to the start of TLA-application, the patient will receive a peripheral intravenous venous catheter (PVC) for additional sedative medication, if necessary.

During TLA-application, the patient must be monitored hemodynamically, e.g., with a pulse oximeter and blood pressure monitors, to immediately detect accidental intravascular infiltration (immediate skin anemia, increase in heart rate) (Table 2).

**Table 2:** (After Roerden [27]). Precautionary and emergency measures

Side effects	Precautionary and emergency measures
systemic toxicity of local anesthetics (LAST)	Termination of the TLA application
	Oxygenation, treatment of seizures (benzodiazepine), resuscitation (if necessary), administration of 20% lipid emulsion
	Continuous monitoring of vital parameters
Neurological symptoms (seizures, unconsciousness, restlessness)	Termination of the TLA application
	Oxygen supply, airway management, treatment of seizures (benzodiazepines), ECG monitoring if necessary
Apnea following cerebral toxicity	Termination of the TLA application
	Oxygen supply, resuscitation, continuous monitoring of vital parameters
Cardiovascular symptoms (bradycardia, hypotension, cardiac arrhythmias)	Termination of the TLA application
	Oxygenation, ECG monitoring
	Treatment according to the ECG findings
Methemoglobinemia	Termination of the TLA application
	Oxygenation
	Application of Methylene Blue
	treatment of seizures (benzodiazepines)
Pneumothorax	Use of non-ground needles in deep areas/ axilla
	Application of TLA in the axilla under sonographic control
	Continuous monitoring of vital parameters
	Chest x-ray
	Chest tube/ needle decompression if necessary

With the above weight-adjusted dosages, one is within the recommended maximum dose, so that even coronary heart disease is not a contraindication for this procedure. With regard to toxic side effects, the absolute dose is less important than the speed of administration.

### Premedication? (Analgo-)Sedation?

Each patient is offered the option of pre- and intraoperative conscious sedation with midazolam (1–5 mg) and is informed of possible side effects.

During surgery, the patient's vital signs are monitored (blood pressure, ECG and oxygen saturation) and documented. In the case of moderate or necessary deep analgosedation, equipment corresponding to an anesthesia workstation must be available that is sufficiently suitable for monitoring and supporting respiratory and cardiovascular monitoring.

All members of the surgical team are regularly trained in emergency situations such as resuscitation and treatment of TLA-associated adverse events (see Table 2). In addition, anesthesiologists are always available for emergencies.

In the case of intraoperative conscious sedation, the patient must still be monitored postoperatively for 2–4 h before being allowed to leave the ward. If sedation is not used, the patient can be discharged 15 min after the procedure.

### Special intraoperative features

Intraoperatively, additional analgesia (e.g. midazolam i.v., piritramide i.v.) can be administered at any time. Injection of a larger volume of 0.21% TLA solution in case of pain is also an option. For mastectomies or partial thoracic muscle resections, additional pectoralis nerve blockades under sonographic view with 10–50 ml of 0.21% TLA may be useful at the beginning of surgery.

Compared to general anesthesia, the surgical field tends to be moister and less bloody due to the tumescent solution. The tissue layers are often easier and gentler to cut with scissors due to hydrodissection. For electrocautery, it is recommended to reduce the standard intensity and perform dissection with scissors or bipolar forceps if there is discomfort in the axilla or prepectoral area.

### Patient selection

The prerequisite for surgery in TLA is a cooperative, willing and understanding patient. Since only the surgical field is anesthetized, the patient can move without restrictions and can be repositioned if necessary.

Mentally unstable patients and those with excessive fear of needles and injections, as well as dementia patients, are not suitable for TLA.

In general, the contraindications are the same as for common local anesthesia.

### First experiences and studies

Initially, fibroadenomas or benign lesions were removed in young patients on an outpatient basis in the TLA*.* Simple segmental resections and mastectomies were also performed in the TLA in older patients in whom the tumor had progressed under anti-hormonal therapy and who had previously refused surgery in general anesthesia.

Based on positive experience and increasing demand we initiated a prospective study including breast cancer patients with the need of surgical removement in TLA versus general anesthesia (Ethics Committee 598/2022BO1). Primary endpoint will be the level of perioperative pain. Secondary endpoints include the health-related quality of life, complications, and perioperative anxiety. Not included will be tumor-adapted reduction mammoplasty, nipple- and skin-sparing mastectomies with implant placement and complete axillary dissections.

## Summary

To date no diagnostic or oncoplastic breast surgery has been routinely performed under tumescent local anesthesia without general anesthesia. This article presents the possible application and the procedure of tumescent local anesthesia for these procedures.

The independence from the anesthesiologist, the low spectrum of side effects compared with general anesthesia as well as good perioperative mobility of the patient are convincing reasons for the use of TLA in breast surgery (Table 3).

**Table 3:** Advantages and disadvantages of TLA

Advantages	Hydrodissection effect
	The addition of epinephrine results in less intraoperative bleeding
	The slow infiltration, pressure buildup, and marked lipophilicity of LA, together with vasoconstrictors, result in a prolonged effect of TLA
	The long-lasting anesthetic effect leads to a reduction in postoperative pain
	Ease of administration—compared to epidural, intercostal block and paravertebral block, TLA is very resource efficient and does not require individuals with special skills
	Local anesthetics also have antimicrobial activity [28]
Disadvantages	Moist surgical site
	Time-consuming infiltration
	Guidance of the awake patient
	Possible intraoperative pain with necessary additional analgesia
	(Low) risk of intravascular application of TLA solution

Tumescent solutions can be easily prepared and gently applied with infusion or roller pumps.

Selecting the correct TLA concentration, cannula, cannula position, flow rate, and volume required requires experience and a learning curve.
